# Observational Study of the Association between Atrial Fibrillation and In-Hospital Mortality during Hospitalization for Solid Organ Transplants in Spain from 2004 to 2021

**DOI:** 10.3390/jcm12227056

**Published:** 2023-11-13

**Authors:** José M de-Miguel-Yanes, Ana Lopez-de-Andres, Rodrigo Jimenez-Garcia, José J Zamorano-Leon, David Carabantes-Alarcon, Valentín Hernández-Barrera, Javier De-Miguel-Diez, Francisco Carricondo, Barbara Romero-Gomez, Natividad Cuadrado-Corrales

**Affiliations:** 1 Internal Medicine Department, Hospital General Universitario Gregorio Marañón, Instituto de Investigación Sanitaria Gregorio Marañón (IiSGM), Universidad Complutense de Madrid, 28040 Madrid, Spain; josemaria.demiguel@salud.madrid.org; 2 Department of Public Health and Maternal & Child Health, Faculty of Medicine, Universidad Complutense de Madrid, 28040 Madrid, Spain; rodrijim@ucm.es (R.J.-G.); josejzam@ucm.es (J.J.Z.-L.); dcaraban@ucm.es (D.C.-A.); mariancu@ucm.es (N.C.-C.); 3 Preventive Medicine and Public Health Teaching and Research Unit, Health Sciences Faculty, Rey Juan Carlos University, Alcorcón, 28922 Madrid, Spain; valentin.hernandez@urjc.es; 4 Respiratory Department, Hospital General Universitario Gregorio Marañón, Facultad de Medicina, Instituto de Investigación Sanitaria Gregorio Marañón (IiSGM), Universidad Complutense de Madrid, 28009 Madrid, Spain; javier.miguel@salud.madrid.org; 5 Laboratory of Neurobiology of Hearing (UCM 910915), Ophthalmology and Otorhinolaryngology, Department of Immunology, Faculty of Medicine, University Complutense, IdISSC, 28040 Madrid, Spain; fjcarric@ucm.es (F.C.); brgomez@ucm.es (B.R.-G.)

**Keywords:** kidney transplant, liver transplant, heart transplant, lung transplant, atrial fibrillation, atrial flutter, in-hospital mortality, Spanish National Hospital Discharge Data

## Abstract

(1) Background: We analyzed the association between atrial fibrillation or atrial flutter (AF) and in-hospital mortality (IHM) among patients who underwent solid organ transplants in Spain from 2004 to 2021. (2) Methods: We gathered information from all hospital admissions for lung, liver, kidney, and heart transplants. (3) Results: A total of 71,827 transplants were analyzed (4598 lung transplants; 18,127 liver transplants; 45,262 kidney transplants; and 4734 heart transplants). One third of these were for women. Overall, the prevalence of AF was 6.8% and increased from 5.3% in 2004–2009 to 8.6% in 2016–2021. The highest prevalence of AF was found for heart transplants (24.0%), followed by lung transplants (14.7%). The rates for kidney and liver transplants were 5.3% and 4.1%, respectively. The AF code increased over time for all of the transplants analyzed (*p* < 0.001). The patients’ IHM decreased significantly from 2004–2009 to 2016–2021 for all types of transplants. AF was associated with a higher IHM for all of the types of transplants analyzed, except for heart transplants. (4) Conclusions: The prevalence of AF among patients admitted for solid organ transplants was highest for those who underwent heart transplants. The mortality rate during the patients’ admission for lung, liver, kidney, or heart transplants decreased over time. AF was independently associated with a higher risk of dying in the hospital for those who underwent lung, liver, or kidney transplants.

## 1. Introduction

Spain leads the international rankings in organ donation and transplants [1]. The prognosis of solid organ transplants could be improved with strategies derived from studies designed to provide a better knowledge of the variables associated with the in-hospital mortality (IHM).

Atrial fibrillation and atrial flutter are supraventricular arrhythmias that share clinical characteristics, such as predisposing factors, symptoms, and therapeutical strategies. Occasionally, it is not easy to differentiate them with a surface electrocardiogram. For research purposes, both will hereafter be referred to as atrial fibrillation (AF).

Many studies have analyzed the incidence of AF both in the short and long term after heart transplants [2,3]. In this setting, atrial flutter may be more common than atrial fibrillation [4]. In heart transplants, vagal denervation and pulmonary vein isolation during the surgical procedure result in a low incidence of AF, and many patients return to sinus rhythm with the implanted organ. The appearance of AF after a heart transplant could be more related to clinical frailty, decompensated systemic medical conditions, or acute rejection and would entail poorer outcomes [2]. An interesting study from Darche FF et al. reported a 2.3% incidence rate of AF during the 30-day period after heart transplants [5] and found an association between preoperative AF and late-onset AF and mortality. 

In recipients of kidney transplants, patients with preoperative (“prevalent”) and postoperative or new-onset (“incident”) AF needing hospitalization have been associated with worse outcomes [6,7]. In the case of liver transplants, there is some evidence that prevalent AF is associated with worse outcomes after the procedure [8]. Although there are fewer studies specifically focusing on incident AF after liver transplants, some results have also suggested an association with mortality [9]. Regarding lung transplants, the evidence of the association between preoperative AF and mortality after the procedure is scarce [10], whilst post-procedural AF has consistently been associated with an increased mortality rate [11,12]. 

The relationship between clinical events, like hospital-acquired pneumonia, and IHM among patients undergoing a solid organ transplant has been previously assessed by our group [13]. We have also analyzed the impact of AF on IHM in other clinical conditions [14]. This background encouraged us to investigate the association between AF and IHM among recipients of organ transplants. 

Hence, we intended to examine the prevalence and time trends, from 2004 to 2021, of AF among lung, liver, kidney, and heart transplant recipients. We also aimed to assess the impact of AF on the patients’ IHM after these procedures.

## 2. Materials and Methods

We did a retrospective, observational analysis using the Spanish National Hospital Discharge Data (SNHDD) [15]. The SNHDD is a large, multicentric, national database spanning a long period of time (for this work, the period was 2004–2021), which includes information from over 95% of hospital admissions in Spain. Public hospitals perform most solid organ transplants in our country [16]. The SNHDD used codes of the International Classification of Diseases, Ninth Revision, Clinical Modification (ICD9-CM) until year 2015, when it was changed to the Tenth Revision (ICD10-CM). Within this database, it is possible to collect information about up to 14 diagnoses and 20 procedures carried out during patients’ hospitalization. We brought together data for 18 complete years (2004–2021).

We selected admissions for those aged ≥18 years whenever a lung transplant, kidney transplant, heart transplant, or liver transplant code appeared at any procedural field during a given hospital admission (Table S1).

We identified all episodes of AF using ICD-9-CM codes 427.31 and 427.32 as well as ICD-10-CM code I48.xx for any diagnosis field.

We assessed patients’ comorbid conditions by means of the Charlson Comorbidity Index (CCI) [17], which collects previous diseases. We grouped patients into three categories: high CCI (two or more diseases), medium CCI (one disease), and low CCI (no diseases). 

We collected data about patients’ age, sex, year of admission, specific in-hospital infections (e.g., cytomegalovirus, urinary tract infection, and hospital-acquired pneumonia), complications of the transplanted organs, and leucopenia. For the period of 2016–2021, we added a COVID-19 code (ICD-10-CM code U07.1) (Table S1).

We analyzed IHM and length of hospital stay (LOHS) as independent variables.

Time was divided in three periods of six years each (2004 to 2009, 2010 to 2015, and 2016 to 2021). 

Absolute frequencies and proportions are provided to describe qualitative variables and means or medians with their corresponding standard deviations or interquartile ranges for quantitative variables. The *t*-test or the Mann–Whitney test and the chi-square test were applied for comparison of quantitative and qualitative variables, respectively.

We used multivariable logistic regression analyses to assess the influence of AF, taken as a covariate, on IHM for each type of transplant, providing odds ratios (ORs) with 95% confidence intervals (CIs) to measure the magnitude and significance of the association. 

To further adjust the model, we did a sensitivity analysis accounting for cardiovascular comorbidities as separate items outside the CCI while also including a modified version of the CCI with no cardiovascular comorbidities. This analysis was aimed to elucidate whether AF was a marker of increased IHM driven by established cardiovascular diseases instead of a mortality risk factor in and of itself. 

Stata version 10.1 was the statistical software used.

### Ethical Statement

The SNHDD is an anonymized database publicly accessible by any researcher, so no ethical approval is needed.

## 3. Results

### 3.1. Atrial Fibrillation/Atrial Flutter during Admission in All Transplants Combined

A total of 71,827 transplants were performed in Spain from the year 2004 to the year 2021. Only 33% of these were for women. The mean patient age has significantly increased over time (*p* < 0.001) (Table 1). 

**Figure 1:**
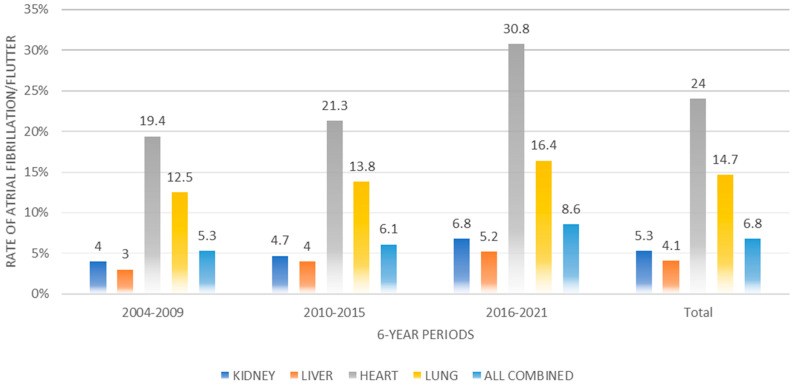
Rates of atrial fibrillation/flutter during admission for solid organ transplants in Spain from year 2004 to year 2021.

The overall prevalence of AF increased from 5.3% in the 2004–2009 period to 8.6% in the last period (2016–2021) (*p* < 0.001) (Table 1 and Figure 1). When we combined the data from all the time periods, the highest rates of AF corresponded to heart transplants (24.0%), followed by lung transplants (14.7%) (Figure 1). For all the transplants, the coding for AF increased over time (*p* < 0.001). Comparing data from 2004 to 2009 with those from 2016 to 2021, the AF rates increased from 4% to 6.8% for kidney transplants, from 3% to 5.2% for liver transplants, from 19.4% to 30.8% for heart transplants, and from 12.5% to 16.4% for lung transplants (all with *p* values < 0.001) (Table 1 and Figure 1).

### 3.2. Atrial Fibrillation/Atrial Flutter during Admission for Kidney Transplants

A total of 45,262 kidney transplants (36% for female patients) were performed in Spain from 2004 to 2021 (Table 2). Age and comorbidity increased over time among these patients (*p* < 0.001). The prevalence of AF was higher among men than women (*p* < 0.001). Patients with AF more frequently suffered kidney transplant complications, urinary tract infections, cytomegalovirus infections, and hospital-acquired pneumonia (all with *p* values ≤0.001). The median LOHS was larger in people with AF (16 ± 15 days vs. 13 ± 10 days; *p* < 0.001). The IHM was also higher in patients with AF (4.0% vs. 1.2%; *p* < 0.001) and remained stable over time among people with AF (*p* = 0.08).

### 3.3. Atrial Fibrillation/Atrial Flutter during Admission for Liver Transplants

A total of 18,127 liver transplants (26.8% for women) were performed in Spain over the study period (Table 3). AF was more prevalent among men, older people, and people with a higher CCI (all *p* values ≤0.001). We detected no significant differences in the LOHS after liver transplants in people with an AF code vs. no AF (*p* = 0.064). The IHM was higher among people with AF (11.7% vs. 7.1%; *p* < 0.001), and there was no significant change over time (*p* = 0.209).

### 3.4. Atrial Fibrillation/Atrial Flutter during Admission for Heart Transplants

From 2004 to 2021, a total of 4734 heart transplants (24.9% for women) were completed in Spain (Table 4). People coded for AF were older and had more comorbidities (*p* values < 0.001), but we could see no sex-associated differences in AF coding (*p* = 0.557). Both the LOHS (*p* = 0.061) and IHM (*p* = 0.485) did not differ between people with vs. without AF. The mortality rate decreased over time among people with AF (from 20.9% in the period of 2004–2009 to 16.5% in the period of 2016–2021; *p* = 0.001).

### 3.5. Atrial Fibrillation/Atrial Flutter during Admission for Lung Transplants

A total of 4598 lung transplants (36.4 % for women) were performed in Spain over the study period (Table 5). People with AF were more often men and older than people without AF (*p* values <0.001). The LOHS was significantly longer in people with AF (39 ± 34 vs. 35 ± 30 days; *p* = 0.015). The IHM was also higher among recipients with AF (17.8% vs. 14.3%; *p* = 0.021) but decreased over time in the population with AF, from 27.8% in the first time period to 15.8% in the last one (*p* = 0.012).

### 3.6. Multivariable Analyis of Varaibles Associated with IHM among Patients Who Underwent Solid Organ Transplants

As can be seen in Table 6, the female sex was associated with a 13% to 24% higher IHM. Also, older patients had an increased risk of IHM among all types of transplant, except for those with lung transplants, and the strongest association was for kidney transplants (OR(CI) = 4.21(3.14–5.65) for people ≥65 years vs. <45 years). Hospital-acquired pneumonia was associated with an increased IHM rate for all the transplants analyzed.

The time trend analysis showed that the IHM was significantly higher in the first period (2004–2009, reference category) decreasing significantly until the last one (2016–2021) for all types of transplants. 

AF was a significant risk factor for IHM for all transplants evaluated, except for heart transplants; the ORs (CIs) for kidney, liver, heart, and lung transplants were 2.17(1.71–2.75), 1.77(1.39–2.26), 1.07(0.89–1.28), and 1.33(1.06–1.67), respectively.

In the sensitivity analysis accounting for cardiovascular comorbidities as separate items outside the CCI, AF remained significantly associated with IHM, with ORs that were similar to those found in the main analyses, despite the strong association between cardiovascular conditions and IHM (Table S2).

## 4. Discussion

We found increasing rates of AF for all of the transplants analyzed. Regrettably, we were not able to differentiate between preoperative vs. postoperative (new-onset) AF, since the ICD-9-CM code did not include a “present on admission” label that would have allowed us to categorize the coded diagnoses as “prevalent” vs. “incident”. Indeed, the distinction between both categories was only possible for the last years of the study period after the transition to the ICD-10-CM code, so we decided not to include this dichotomy to avoid eliciting more complex and possibly confounded results.

The highest values for AF rates corresponded to heart transplants. These figures were probably driven by preoperative AF, although this argument is merely speculative due to the reason mentioned in the paragraph above. The prevalence of AF in stage IV heart failure has been reported to be as high as 50% in older studies [18], but more recent research has reported similar numbers even in less advanced stages of heart failure [19]. We expected to see a relatively high AF rate among people undergoing lung transplants. Early in the postoperative period, the AF rate has been reported to be around 20% for lung transplants [20]. Some specific factors have been associated with this complication after lung transplants: contraindications for pain control with an epidural catheter, underlying pulmonary fibrosis, or procedures resulting in more inflammation of the pericardium or atriums [21].

An AF code was more frequent among men than among women for all types of solid transplants in our study except for heart transplants. Sadly, the design of our study did not let us give a convincing explanation for this finding. We could hypothesize that when diseases leading to transplantation are intrinsic to the heart, the higher rates of AF associated with heart diseases that are more prevalent among men (e.g., coronary artery disease) are counterbalanced by the higher rates of AF associated with heart diseases that are more prevalent among women (e.g., valvular heart disease). Rates of AF might differ among men and women for other types of solid organ transplants because age and comorbidities predisposing patients to AF (e.g., chronic obstructive pulmonary disease) are generally not well balanced between both sexes [22]. 

The good news is that IHM decreased over time for all the transplants analyzed. Strikingly, not many studies focus on short-term mortality: rather, they tend to evaluate long-term outcomes. The study by Awan AA et al. reported a reduction in mortality during the 12-month period after kidney transplantation in the United States [23]. This reduction in mortality rates after both kidney and liver transplants is more notable in the short term than in the long term [24,25]. A reduction in the number of cardiovascular events after the former [23] and a better control of comorbidities after the latter [26] might explain the decreasing short-term mortality rates described for these two types of transplants. Recent changes in perioperative procedures have been proposed to influence short-term mortality rates after heart [27] and lung transplants [28].

We found that being of an advanced age was a risk factor for IHM among all transplants except for lung transplants in the multivariate analyses. Adjusting for the full set of variables attenuated the association seen in the bivariate analyses between age and IHM in the case of lung transplants. This seems to counter previous knowledge about this type of transplant [29]. The reason for this could be the low number of lung transplants performed in the population group aged ≥65 years (less than 10% of the total, the lowest percentage among all transplants). This relatively low percentage probably is due to an optimal selection of older candidates to pursue lower mortality rates [30] and there may be less statistical power to detect a difference in the opposite direction. 

IHM was higher among people undergoing kidney, liver, or lung transplants who were coded for AF vs. that of people who were not coded for AF in both the bivariate and multivariate analyses. As the main novelty of our study, we found no differences in IHM among people coded for AF vs. that of people who were not coded for AF during their admission for heart transplants. In the case of kidney [6,31,32], liver [8], and lung transplants [33], several studies have reached similar conclusions, although conflicting results have also been published, for example, for liver transplants [34]. In the case of heart transplants, a meta-analysis by Chokesuwattanaskul R et al. reported a pooled OR (95%CI) = 2.86(2.08–3.93) for mortality among those with AF compared to those patients without this condition [35]. Contrary to this and to other papers [5], our results are in concordance with a recent study from Isath A et al. who reported similar rates of IHM among people undergoing heart transplantation who developed postoperative AF vs. those without AF [36]. These authors ascribe their findings to improvements in post-transplant care including better arrhythmia treatment and monitoring as well as more recent immunosuppressive medications. Moreover, early AF after heart transplantation has been highlighted as a marker of acute rejection. The onset of AF should activate a proactive search of this complication to improve the prognosis of these patients who otherwise will suffer delays in the detection of graft rejections [37]. Nonetheless, we cannot rule out an impact on long-term mortality in people following heart transplants coded for AF [38], since this was not the goal of our research.

Herein, we present the data of almost 72,000 patients who received solid organ transplants and over 4800 AF codes during a 18-year study period. Our database covered over 95% of the population of our country. However, several limitations must be mentioned. First, we did not differentiate between preoperative vs. postoperative AF for the reasons explained earlier in this section. Second, inaccuracies in coding secondary diagnoses are possible, but significant mistakes in coding organ transplants are unlikely. Third, if the improbable situation occurs in which a patient is admitted to two different hospitals after transplantation, they would appear twice. Fourth, residual confounding variables due to variables not considered in our investigation and associated with IHM cannot be ruled out. Fifth, the cause of death is relevant information that unfortunately is not included in the SNHDD database; if possible, future investigations should collect and analyze these data. And sixth, we did not have the possibility of knowing the anticoagulation treatments used in our population. Indications have changed over time (the use of the CHA_2_DS_2_-VASc score instead of the CHADS_2_ score) and the use of new direct oral anticoagulants in this more recent manner may have influenced our results.

## 5. Conclusions

In summary, IHM significantly decreased over time for kidney, liver, heart, and lung transplants in Spain from the year 2004 to the year 2021. The IHM was significantly higher in people coded for AF during their admission for transplants of any solid organ except for heart transplants. Additional studies are needed to understand whether AF influences the short-term outcomes by itself, or rather it is a marker of vulnerability in this setting. A deeper understanding of this association might aid strategies aimed to lower the short-term mortality in this population.

## Figures and Tables

**Table 1:** Distribution according to study variables of patients with and without an atrial fibrillation/flutter (AF) diagnosis who received solid organ transplants in Spain from year 2004 to year 2021.

		2004–2009		2010–2015		2016–2021		TOTAL		*p* Values	
		No AF	AF	No AF	AF	No AF	AF	No AF	AF	AF vs. No AF	AF Trend
* Number of transplants, n		20,391	1130	21,629	1403	24,924	2350	66,944	4883	<0.001	<0.001
Male sex, n (%)		13,458(66)	793(70.2)	14,498(67.0)	1026(73.1)	16,594(66.6)	1739(74.0)	44,550(66.6)	3558(72.9)	<0.001	<0.001
Female sex, n (%)		6933(34.0)	337(29.8)	7131(33.0)	377(26.9)	8330(33.4)	611(26.0)	22,394(33.5)	1325(27.1)	<0.001	<0.001
Age, mean (SD)		49.2(15.4)	58.0(10.7)	51.5(14.9)	60.2(10.0)	53.4(16.0)	61.7(10.4)	51.5(15.4)	60.4(10.4)	<0.001	<0.001
Age <45 years, n (%)		6438(31.6)	103(9.1)	5606(25.9)	89(6.3)	5583(22.4)	137(5.8)	17,627(26.3)	329(6.7)	<0.001	0.001
Age 45–54 years, n (%)		5216(25.6)	243(21.5)	5581(25.8)	233(16.6)	5357(21.5)	293(12.5)	16,154(24.1)	769(15.8)	<0.001	0.060
Age 55–64 years, n (%)		6002(29.4)	492(43.5)	6584(30.4)	593(42.3)	8039(32.3)	923(39.3)	20,625(30.8)	2008(41.1)	<0.001	<0.001
Age ≥ 65 years, n (%)		2735(13.4)	292(25.8)	3858(17.8)	488(34.8)	5945(23.9)	997(42.4)	12,538(18.7)	1777(36.4)	<0.001	<0.001
CCI, mean (SD)		2.6(1.3)	2.4(1.4)	2.7(1.4)	2.5(1.4)	2.8(1.5)	2.8(1.6)	2.7(1.4)	2.6(1.5)	0.003	<0.001
Low CCI, n (%)		709(3.5)	56(5.0)	694(3.2)	74(5.3)	1026(4.1)	136(5.8)	2429(3.6)	266(5.5)	<0.001	0.002
Medium CCI, n (%)		11,690(57.3)	536(47.4)	11,818(54.6)	622(44.3)	12,188(49.0)	775(33.0)	35,696(53.3)	1933(40.0)	<0.001	<0.001
High CCI, n (%)		7992(39.2)	538(47.6)	9117(42.2)	707(50.4)	11,710(47.0)	1439(61.2)	28,819(43.1)	2684(55.0)	<0.001	<0.001
¶ Complications	Yes	5912(29.0)	393(34.8)	6123(28.3)	474(33.8)	6995(28.1)	809(34.4)	19,030(28.4)	1676(34.3)	<0.001	<0.001
¶ Urinary tract infection	Yes	2015(9.9)	119(10.5)	2367(10.9)	160(11.4)	2409(9.7)	248(10.6)	6791(10.1)	527(10.8)	0.148	<0.001
¶ Cytomegalovirus	Yes	354(1.7)	31(2.7)	420(1.9)	39(2.8)	742(3.0)	99(4.2)	1516(2.3)	169(3.5)	<0.001	0.026
¶ Leucopenia	Yes	52(0.3)	4(0.4)	231(1.1)	18(1.3)	179(0.7)	26(1.1)	462(0.7)	48(1.0)	0.019	0.066
¶ COVID-19	Yes	NA	NA	NA	NA	74(0.3)	8(0.34)	74(0.1)	8(0.2)	0.287	NA
¶ Pneumonia (HAP)	Yes	588(2.9)	52(4.6)	516(2.4)	52(3.7)	556(2.2)	110(4.7)	1660(2.5)	214(4.4)	<0.001	<0.001
LOHS, median (IQR)		17(16)	23(28)	16(15)	21(22)	15(15)	21(24)	16(15)	22(24)	<0.001	0.005
IHM, n (%)		1103(5.4)	155(13.7)	867(4.0)	128(9.1)	867(3.5)	208(8.9)	2837(4.2)	491(10.1)	<0.001	<0.001

AF: Atrial fibrillation/flutter. SD: Standard deviation. CCI: Charlson Comorbidity Index. HAP: Hospital-acquired pneumonia. LOHS: Length of hospital stay. IQR: Interquartile range. IHM: In-hospital mortality. NA: Not applicable. *: percentages for this row are shown in Figure 1. ¶: data expressed as n (%).

**Table 2:** Distribution according to study variables of patients with and without an atrial fibrillation/flutter (AF) diagnosis who received kidney transplants in Spain from year 2004 to year 2021.

		2004–2009		2010–2015		2016–2021		Total		*p* Values	
		No AF	AF	No AF	AF	No AF	AF	No AF	AF	AF vs. No AF	AF Trend
* Number of transplants, n		12,466	514	13,776	672	16,627	1207	42,869	2393	<0.001	<0.001
Male sex, n (%)		7812(62.7)	324(63.0)	8777(63.7)	457(68.0)	10,730(64.5)	879(72.8)	27,319(63.7)	1660(69.4)	<0.001	<0.001
Female sex, n (%)		4654(37.3)	190(37.0)	4999(36.3)	215(32.0)	5897(35.5)	328(27.2)	15,550(36.3)	733(30.6)	<0.001	<0.001
Age, mean (SD)		49.0(15.2)	60.7(9.6)	51.6(15.0)	64.0(8.2)	53.9(15.4)	65.8(8.5)	51.7(15.3)	64.2(8.9)	<0.001	<0.001
Age <45 years, n (%)		4455(35.7)	30(5.8)	4061(29.5)	13(1.9)	4009(24.1)	21(1.7)	12,525(29.2)	64(2.7)	<0.001	0.309
Age 45–54 years, n (%)		2980(23.9)	92(17.9)	3348(24.3)	76(11.3)	3657(22.0)	96(8.0)	9985(23.3)	264(11.0)	<0.001	0.299
Age 55–64 years, n (%)		3064(24.6)	195(37.9)	3442(25.0)	227(33.8)	4413(26.5)	349(28.9)	10,919(25.5)	771(32.2)	<0.001	0.013
Age ≥ 65 years, n (%)		1967(15.8)	197(38.3)	2925(21.2)	356(53.0)	4548(27.4)	741(61.4)	9440(22.0)	1294(54.1)	<0.001	<0.001
CCI, mean (SD)		2.5(0.9)	2.7(1.0)	2.7(1.1)	2.9(1.1)	2.8(1.2)	3.2(1.3)	2.7(1.1)	3.0(1.2)	<0.001	<0.001
Low CCI, n (%)		123(1.0)	7(1.4)	74(0.5)	6(0.9)	177(1.1)	5(0.4)	374(0.9)	18(0.8)	0.537	0.235
Medium CCI, n (%)		8183(65.6)	273(53.1)	8484(61.6)	320(47.6)	9074(54.6)	449(37.2)	25,741(60.1)	1042(43.5)	<0.001	<0.001
High CCI, n (%)		4160(33.4)	234(45.5)	5218(37.9)	346(51.5)	7376(44.4)	753(62.4)	16,754(39.1)	1333(55.7)	<0.001	<0.001
¶ Complications	Yes	3415(27.4)	168(32.7)	3625(26.3)	212(31.6)	4265(25.7)	397(32.9)	11,305(26.4)	777(32.5)	<0.001	<0.001
¶ Urinary tract infection	Yes	1704(13.7)	97(18.9)	1991(14.5)	123(18.3)	2027(12.2)	189(15.7)	5722(13.4)	409(17.1)	<0.001	<0.001
¶ Cytomegalovirus	Yes	127(1.0)	9(1.8)	159(1.2)	15(2.2)	348(2.1)	31(2.6)	634(1.5)	55(2.3)	0.001	0.643
¶ Leucopenia	Yes	35(0.3)	2(0.4)	152(1.1)	9(1.3)	128(0.8)	16(1.3)	315(0.7)	27(1.1)	0.031	0.094
¶ COVID-19	Yes	NA	NA	NA	NA	43(0.3)	6(0.5)	43(0.1)	6(0.3)	0.029	NA
¶ Pneumonia (HAP)	Yes	136(1.1)	8(1.6)	148(1.1)	9(1.3)	153(0.9)	32(2.7)	437(1.0)	49(2.1)	<0.001	<0.001
LOHS, median (IQR)		14(12)	18(17)	13(10)	16(14)	13(10)	16(14)	13(10)	16(15)	<0.001	0.002
IHM, n (%)		173(1.4)	25(4.9)	139(1.0)	25(3.7)	195(1.2)	45(3.7)	507(1.2)	95(4.0)	<0.001	0.080

AF: Atrial fibrillation/flutter. SD: Standard deviation. CCI: Charlson Comorbidity Index. HAP: Hospital-acquired pneumonia. LOHS: Length of hospital stay. IQR: Interquartile range. IHM: In-hospital mortality. NA: Not applicable. *: percentages for this row are shown in Figure 1. ¶: data expressed as n (%).

**Table 3:** Distribution according to study variables of patients with and without an atrial fibrillation/flutter (AF) diagnosis who received liver transplants in Spain from year 2004 to year 2021.

		2004–2009		2010–2015		2016–2021		TOTAL		*p* Values	
		No AF	AF	No AF	AF	No AF	AF	No AF	AF	AF vs. No AF	AF Trend
* Number of transplants, n		5890	181	5630	235	5871	320	17,391	736	<0.001	<0.001
Male sex, n (%)		4190(71.1)	145(80.1)	4205(74.7)	191(81.3)	4291(73.1)	254(79.4)	12,686(73.0)	590(80.2)	<0.001	<0.001
Female sex, n (%)		1700(28.9)	36(19.9)	1425(25.3)	44(18.7)	1580(26.9)	66(20.6)	4705(27.1)	146(19.8)	<0.001	0.001
Age, mean (SD)		50.0(15.3)	59.5(8.2)	51.7(14.7)	59.8(7.2)	53.3(15.6)	60.4(8.7)	51.7(15.3)	60.0(8.1)	<0.001	0.430
Age <45 years, n (%)		1380(23.4)	4(2.2)	981(17.4)	9(3.8)	918(15.6)	10(3.1)	3279(18.9)	23(3.1)	<0.001	0.024
Age 45–54 years, n (%)		1746(29.6)	47(26.0)	1753(31.1)	37(15.7)	1271(21.7)	35(10.9)	4770(27.4)	119(16.2)	<0.001	0.986
Age 55–64 years, n (%)		2183(37.1)	82(45.3)	2197(39.0)	128(54.5)	2598(44.3)	168(52.5)	6978(40.1)	378(51.4)	<0.001	<0.001
Age ≥ 65 years, n (%)		581(9.9)	48(26.5)	699(12.4)	61(26.0)	1084(18.5)	107(33.4)	2364(13.6)	216(29.4)	<0.001	0.295
CCI, mean (SD)		3.1(1.8)	3.5(1.8)	3.4(1.8)	3.5(1.6)	3.4(1.9)	3.8(1.9)	3.3(1.9)	3.6(1.8)	0.001	0.084
Low CCI, n (%)		248(4.2)	3(1.7)	220(3.9)	4(1.7)	349(5.9)	11(3.4)	817(4.7)	18(2.5)	0.004	0.119
Medium CCI, n (%)		2491(42.3)	58(32.0)	2232(39.6)	82(34.9)	1961(33.4)	84(26.3)	6684(38.4)	224(30.4)	<0.001	<0.001
High CCI, n (%)		3151(53.5)	120(66.3)	3178(56.5)	149(63.4)	3561(60.7)	225(70.3)	9890(56.9)	494(67.1)	<0.001	<0.001
¶ Complications	Yes	1688(28.7)	45(24.9)	1531(27.2)	56(23.8)	1512(25.8)	86(26.9)	4731(27.2)	187(25.4)	0.283	<0.001
¶ Urinary tract infection	Yes	247(4.2)	8(4.4)	292(5.2)	17(7.2)	260(4.4)	26(8.1)	799(4.6)	51(6.9)	0.003	0.004
¶ Cytomegalovirus	Yes	175(3.0)	5(2.8)	199(3.5)	8(3.4)	271(4.6)	20(6.3)	645(3.7)	33(4.5)	0.278	0.040
¶ Leucopenia	Yes	12(0.2)	0(0)	57(1.0)	2(0.9)	47(0.8)	6(1.9)	116(0.7)	8(1.1)	0.176	0.074
¶ COVID-19	Yes	NA	NA	NA	NA	23(0.4)	2(0.6)	23(0.1)	2(0.3)	0.318	NA
¶ Pneumonia (HAP)	Yes	295(5.0)	9(5.0)	197(3.5)	7(3.0)	160(2.7)	16(5.0)	652(3.8)	32(4.4)	0.404	0.005
LOHS, median (IQR)		21(20)	22(24)	20(19)	21(19)	18(17)	20(22)	20(18)	21(21)	0.064	0.521
IHM, n (%)		503(8.5)	30(16.6)	378(6.7)	26(11.1)	359(6.1)	30(9.4)	1240(7.1)	86(11.7)	<0.001	0.209

AF: Atrial fibrillation/flutter. SD: Standard deviation. CCI: Charlson Comorbidity Index. HAP: Hospital-acquired pneumonia. LOHS: Length of hospital stay. IQR: Interquartile range. IHM: In-hospital mortality. NA: Not applicable. *: percentages for this row are shown in Figure 1. ¶: data expressed as n (%).

**Table 4:** Distribution according to study variables of patients with and without an atrial fibrillation/flutter (AF) diagnosis who received heart transplants in Spain from year 2004 to year 2021.

		2004–2009		2010–2015		2016–2021		TOTAL		*p* Values	
		No AF	AF	No AF	AF	No AF	AF	No AF	AF	AF vs. No AF	AF Trend
* Number of transplants, n		1328	320	1125	305	1146	510	3599	1135	<0.001	<0.001
Male sex, n (%)		1032(77.7)	246(76.9)	849(75.5)	237(77.7)	814(71.0)	378(74.1)	2695(74.9)	861(75.9)	0.557	<0.001
Female sex, n (%)		296(22.3)	74(23.1)	276(24.5)	68(22.3)	332(29.0)	132(25.9)	904(25.1)	274(24.1)	0.557	0.003
Age, mean (SD)		49.4(17.3)	54.3(12.1)	49.5(16.9)	54.5(11.9)	46.1(19.5)	54.4(11.9)	48.4(18.0)	54.4(11.9)	<0.001	0.992
Age <45 years, n (%)		352(26.5)	54(16.9)	307(27.3)	50(16.4)	392(34.2)	87(17.1)	1051(29.2)	191(16.8)	<0.001	0.042
Age 45–54 years, n (%)		324(24.4)	76(23.8)	253(22.5)	82(26.9)	238(20.8)	116(22.8)	815(22.7)	274(24.1)	0.296	<0.001
Age 55–64 years, n (%)		485(36.5)	148(46.3)	406(36.1)	119(39.0)	364(31.8)	220(43.1)	1255(34.9)	487(42.9)	<0.001	<0.001
Age ≥ 65 years, n (%)		167(12.6)	42(13.1)	159(14.1)	54(17.7)	152(13.3)	87(17.1)	478(13.3)	183(16.1)	0.016	<0.001
CCI, mean (SD)		1.7(1.1)	1.9(1.1)	1.8(1.2)	1.9(1.2)	2.2(1.4)	2.7(1.5)	1.9(1.3)	2.2(1.4)	<0.001	<0.001
Low CCI, n (%)		102(7.7)	12(3.8)	71(6.3)	16(5.3)	65(5.7)	15(2.9)	238(6.6)	43(3.8)	<0.001	0.101
Medium CCI, n (%)		555(41.8)	135(42.2)	458(40.7)	119(39.0)	337(29.4)	98(19.2)	1350(37.5)	352(31.0)	<0.001	0.239
High CCI, n (%)		671(50.5)	173(54.1)	596(53.0)	170(55.7)	744(64.9)	397(77.8)	2011(55.9)	740(65.2)	<0.001	<0.001
¶ Complications	Yes	360(27.1)	116(36.3)	395(35.1)	96(31.5)	394(34.4)	171(33.5)	1149(31.9)	383(33.7)	0.253	0.019
¶ Urinary tract infection	Yes	59(4.4)	11(3.4)	68(6.0)	14(4.6)	115(10.0)	30(5.9)	242(6.7)	55(4.9)	0.023	0.348
¶ Cytomegalovirus	Yes	28(2.1)	13(4.1)	39(3.5)	11(3.6)	76(6.6)	31(6.1)	143(4.0)	55(4.9)	0.200	0.947
¶ Leucopenia	Yes	1(0.1)	0(0)	7(0.6)	2(0.7)	5(0.4)	5(1.0)	13(0.4)	7(0.6)	0.247	0.156
¶ COVID-19	Yes	NA	NA	NA	NA	5(0.4)	0(0)	5(0.1)	0(0)	0.209	NA
¶ Pneumonia (HAP)	Yes	69(5.2)	18(5.6)	63(5.6)	17(5.6)	100(8.7)	33(6.5)	232(6.5)	68(6.0)	0.583	0.455
LOHS, median (IQR)		26(30)	30(31)	29(37)	27(29)	37(49)	32(36)	30(39)	30(34)	0.061	0.229
IHM, n (%)		238(17.9)	67(20.9)	202(18.0)	48(15.7)	159(13.9)	84(16.5)	599(16.6)	199(17.5)	0.485	0.001

AF: Atrial fibrillation/flutter. SD: Standard deviation. CCI: Charlson Comorbidity Index. HAP: Hospital-acquired pneumonia. LOHS: Length of hospital stay. IQR: Interquartile range. IHM: In-hospital mortality. NA: Not applicable. *: percentages for this row are shown in Figure 1. ¶: data expressed as n (%).

**Table 5:** Distribution according to study variables of patients with and without an atrial fibrillation/flutter (AF) diagnosis who received lung transplants in Spain from year 2004 to year 2021.

		2004–2009		2010–2015		2016–2021		TOTAL		*p* Values	
		No AF	AF	No AF	AF	No AF	AF	No AF	AF	AF vs. No AF	AF Trend
* Number of transplants, n		881	126	1263	202	1778	348	3922	676	<0.001	0.002
Male sex, n (%)		546(62.0)	89(70.6)	776(61.4)	150(74.3)	1110(62.4)	255(73.3)	2432(62.0)	494(73.1)	<0.001	0.008
Female sex, n (%)		335(38.0)	37(29.4)	487(38.6)	52(25.7)	668(37.6)	93(26.7)	1490(38.0)	182(26.9)	<0.001	0.175
Age, mean (SD)		46.5(15.5)	54.2(10.0)	51.0(13.7)	56.4(9.2)	53.0(13.6)	58.7(7.8)	50.9(14.3)	57.1(8.8)	<0.001	<0.001
Age <45 years, n (%)		302(34.3)	15(11.9)	300(23.8)	17(8.4)	366(20.6)	21(6.0)	968(24.7)	53(7.8)	<0.001	0.687
Age 45–54 years, n (%)		217(24.6)	33(26.2)	282(22.3)	39(19.3)	313(17.6)	54(15.5)	812(20.7)	126(18.6)	0.219	0.528
Age 55–64 years, n (%)		331(37.6)	69(54.8)	594(47.0)	126(62.4)	860(48.3)	206(59.2)	1785(45.5)	401(59.3)	<0.001	0.281
Age ≥ 65 years, n (%)		31(3.5)	9(7.1)	87(6.9)	20(9.9)	239(13.4)	67(19.3)	357(9.1)	96(14.2)	<0.001	0.772
CCI, mean (SD)		1(1.0)	1.0(0.8)	1.0(0.9)	1.2(1.2)	1.1(1.0)	1.1(1.0)	1.0(1.0)	1.1(1.0)	0.183	0.086
Low CCI, n (%)		240(27.2)	34(27.0)	337(26.7)	48(23.8)	465(26.2)	107(30.8)	1042(26.6)	189(28.0)	0.451	0.007
Medium CCI, n (%)		496(56.3)	72(57.1)	676(53.5)	103(51.0)	948(53.3)	151(43.4)	2120(54.1)	326(48.2)	0.005	0.539
High CCI, n (%)		145(16.5)	20(15.9)	250(19.8)	51(25.3)	365(20.5)	90(25.9)	760(19.4)	161(23.8)	0.008	0.028
¶ Complications	Yes	414(47.0)	62(49.2)	560(44.3)	102(50.5)	796(44.8)	153(44.0)	1770(45.1)	317(46.9)	0.395	0.141
¶ Urinary tract infection	Yes	22(2.5)	3(2.4)	35(2.8)	7(3.5)	53(3.0)	8(2.3)	110(2.8)	18(2.7)	0.836	0.984
¶ Cytomegalovirus	Yes	27(3.1)	4(3.2)	28(2.2)	6(3.0)	77(4.3)	20(5.8)	132(3.4)	30(4.4)	0.163	0.336
¶ Leucopenia	Yes	5(0.6)	2(1.6)	17(1.4)	5(2.5)	7(0.4)	0(0)	29(0.7)	7(1.0)	0.420	0.188
¶ COVID-19	Yes	NA	NA	NA	NA	4(0.2)	0(0)	4(0.1)	0(0)	0.406	NA
¶ Pneumonia (HAP)	Yes	95(10.8)	18(14.3)	115(9.1)	20(9.9)	154(8.7)	29(8.3)	364(9.3)	67(9.9)	0.604	0.985
LOHS, median (IQR)		35(33)	47(44)	36(31)	38(36)	34(29)	38(30)	35(30)	39(34)	0.015	0.053
IHM, n (%)		211(24.0)	35(27.8)	167(13.2)	30(14.9)	184(10.4)	55(15.8)	562(14.3)	120(17.8)	0.021	0.012

AF: Atrial fibrillation/flutter. SD: Standard deviation. CCI: Charlson Comorbidity Index. HAP: Hospital-acquired pneumonia. LOHS: Length of hospital stay. IQR: Interquartile range. IHM: In-hospital mortality. NA: Not applicable. *: percentages for this row are shown in Figure 1. ¶: data expressed as n (%).

**Table 6:** Result of multivariable logistic regression analysis to identify study variables independently associated with IMH according to organ transplants.

		Kidney	Liver	Heart	Lung	All
		OR (95% CI)	OR (95% CI)	OR (95% CI)	OR (95% CI)	OR (95% CI)
Sex	Female	1.24(1.04–1.47)	1.13(0.99–1.28)	1.24(1.04–1.48)	1.16(0.97–1.38)	1.08(1.00–1.17)
Age groups	<45 years	1	1	1	1	1
	45–54 years	2.05(1.49–2.83)	0.9(0.75–1.08)	1.01(0.80–1.28)	1.17(0.91–1.51)	1.18(1.06–1.32)
	55–64 years	2.57(1.90–3.47)	1.01(0.85–1.20)	1.38(1.12–1.69)	1.11(0.89–1.39)	1.55(1.40–1.71)
	≥65 years	4.21(3.14–5.65)	1.55(1.27–1.90)	1.41(1.09–1.82)	0.90(0.63–1.30)	1.36(1.21–1.53)
Complications		2.20(1.86–2.60)	3.24(2.88–3.64)	1.13(0.96–1.33)	1.12(0.94–1.32)	2.21(2.05–2.37)
Urinary tract infection		0.41(0.30–0.55)		0.65(0.45–0.94)		0.40(0.34–0.47)
Cytomegalovirus		2.07(1.36–3.14)				
Leucopenia				2.59(1.01–6.66)		
Hospital acquired pneumonia		5.03(2.56–10.32)	3.16(2.58–3.88)	2.30(1.76–3.01)	2.73(2.16–3.45)	4.25(3.66–4.92)
Year of transplant	2004–2009	1	1	1	1	1
	2010–2015	0.65(0.53–0.81)	0.80(0.70–0.92)	0.92(0.76–1.11)	0.47(0.38–0.58)	0.71(0.65–0.78)
	2016–2021	0.63(0.52–0.77)	0.72(0.62–0.83)	0.73(0.60–0.89)	0.38(0.31–0.47)	0.59(0.54–0.64)
Atrial fibrillation/atrial flutter		2.17(1.71–2.75)	1.77(1.39–2.26)	1.07(0.89–1.28)	1.33(1.06–1.67)	2.17(1.96–2.42)

OR: Odds ratio. 95% CI: 95% confidence interval.

## Data Availability

According to the contract signed with the Spanish Ministry of Health and Social Services, which provided access to the databases from the Spanish National Hospital Database (Registro de Actividad de Atención Especializada, Conjunto Mínimo Básico de Datos: Registry of Specialized Health Care Activities, Minimum Basic Data Set), we cannot share the databases with any other investigator, and we have to destroy the databases once the investigation has concluded. Consequently, we cannot upload the databases to any public repository. However, any investigator can apply for access to the databases by filling out the questionnaire available at https://www.sanidad.gob.es/estadEstudios/estadisticas/estadisticas/estMinisterio/SolicitudCMBD.htm (accessed on 20 August 2023). All other relevant data are included in the paper.

## Supplementary Materials

The following supporting information can be downloaded at: https://www.mdpi.com/article/10.3390/jcm12227056/s1, Table S1: ICD-9-CM and ICD-10-CM codes for diagnoses and procedures. Table S2: Multivariate analysis of the factors associated with in-hospital mortality among patients who underwent a solid organ transplant in Spain from 2004 to 2021 including the cardiovascular diseases of the Charlson Comorbidity Index.
