# Supplementary material for: Observational Study of the Association between Atrial Fibrillation and In-Hospital Mortality during Hospitalization for Solid Organ Transplants in Spain from 2004 to 2021
Source: J Clin Med. 2023 Nov 13;12(22):7056. doi: 10.3390/jcm12227056 (PMC10671923; doi:10.3390/jcm12227056)
Supplement: Supplementary file 1 [file jcm-12-07056-s001.zip › jcm-2668575-supplementary.pdf]

**Table S1**. ICD-9-CM and ICD-10-CM codes for diagnoses and procedures used in this investigation.

| | | ICD 9 | ICD 10 |
|---|---|---|---|
| **ATRIAL FIBRILLATION/FLUTTER** | | | |
| | Diagnoses | 427.31<br>427.32 | I48 |
| **KIDNEY TRANSPLANT** | | | |
| | Procedures | 55.6, 55.61, 55.69 | 0TYxxxx |
| | Diagnoses | 996.81 | T86.1, T86.10, T86.11, T86.12, T86.13, T86.19 |
| **LIVER TRANSPLANT** | | | |
| | Procedures | 50.5, 50.51, 50.59 | 0FY0 |
| | Diagnoses | 996.82 | T86.4, T86.40, T86.41, T86.42, T86.43, T86.49 |
| **HEART TRANSPLANT** | | | |
| | Procedures | 37.51 | 02YA0Zx |
| | Diagnoses | 996.83 | T86.2 , T86.20, T86.21, T86.22, T86.23, T86.29, T86.290, T86.298 |
| **LUNG TRANSPLANT** | | | |
| | Procedures | 33.50, 33.51, 33.52 | 0BYC, 0BYD, 0BYF, 0BYG, 0BYH, 0BYJ, 0BYK 0BYL, 0BYM |
| | Diagnoses | 996.84 | T86.81, T86.810, T86.10, T86.811, T86.812, T86.813, T86.819 |
| **CYTOMEGALOVIRUS INFECTION** | | 078.5 | B25.x |
| **PNEUMONIA** | | 481-486<br>997.31 | J13-J18,<br>J95.851 |
| **URINARY TRACT INFECTION** | | 599.0 | N39.0 |
| **LEUCOPENIA** | | 288.50, 288.51, 288.59 | D72.810, D72.818, D72.819 |
| **CIRRHOSIS** | | 571.2, 571.5, 571.6 | K70.3x, K71.7, P78.81, K74.3, K74.4, K74.5, K74.6x |
| **HOSPITAL-ACQUIRED PNEUMONIA** | | 481-486, 997.31 | JI3-JI8, J95.851 |
| **COVID-19** | | | U07.1 |

**Table S2.** Multivariate analysis of the factors associated with in-hospital mortality among patients who underwent a solid organ transplant in Spain from 2004 to 2021 including the cardiovascular diseases of the Charlson Comorbidity Index

| | | KIDNEY<br>OR (95% CI) | LIVER<br>OR (95% CI) | HEART<br>OR (95% CI) | LUNG<br>OR (95% CI) | ALL<br>OR (95% CI) |
|---|---|---|---|---|---|---|
| Sex | Female | 1.29(1.09-1.53) | 1.09(0.95-1.23) | 1.24(1.04-1.48) | 1.14(0.96-1.37) | 1.12(1.04-1.21) |
| Age groups | <45 years | 1 | 1 | 1 | 1 | 1 |
| | 45-54 years | 2.05(1.48-2.83) | 0.92(0.77-1.1) | 1(0.79-1.27) | 1.2(0.93-1.55) | 1.24(1.1-1.38) |
| | 55-64 years | 2.54(1.88-3.44) | 1.04(0.88-1.23) | 1.36(1.1-1.67) | 1.16(0.92-1.46) | 1.64(1.48-1.82) |
| | ≥ 65 years | 4.15(3.09-5.58) | 1.59(1.3-1.94) | 1.41(1.08-1.83) | 0.95(0.66-1.37) | 1.54(1.37-1.74) |
| Myocardial infarction | | 3,17(2,35-4,28) | 2.97(1.93-4.58) | | 4.15(2.24-7.69) | 1.83(1.58-2.12) |
| Congestive cardiac failure | | 3,14(2,47-3,99) | 3.05(2.35-3.94) | | 2.69(1.95-3.72) | 2.51(2.29-2.77) |
| Cerebral vascular disease | | 1.9(1.24-2.9) | 3.99(2.85-5.59) | 1.5(1.11-2.01) | 2.83(1.93-4.15) | 2.49(2.1-2.96) |
| Complications | | 2.2(1.86-2.6) | 3.27(2.91-3.68) | 1.15(0.98-1.36) | 1.1(0.93-1.31) | 2.23(2.07-2.4) |
| Urinary tract infection | | 0.41(0.3-0.56) | | 0.64(0.44-0.92) | | 0.4(0.34-0.48) |
| Cytomegalovirus | | 2.07(1.36-3.14) | | | | |
| Leucopenia | | | | 2.51(1.01-6.33) | | |
| Hospital acquired pneumonia | | 5.28(2.65-11.32) | 3.04(2.47-3.73) | 2.23(1.7-2.92) | 2.64(2.08-3.34) | 4.65(4.11-5.26) |
| Year of transplant | 2004-2009 | 1 | 1 | 1 | 1 | 1 |
| | 2010-2015 | 0.64(0.52-0.79) | 0.8(0.7-0.92) | 0.92(0.76-1.11) | 0.47(0.38-0.58) | 0.72(0.66-0.78) |
| | 2016-2021 | 0.6(0.49-0.74) | 0.71(0.62-0.82) | 0.73(0.6-0.88) | 0.37(0.3-0.45) | 0.61(0.55-0.66) |
| Atrial fibrillation/atrial flutter | | 2.02(1.59-2.56) | 1.64(1.28-2.09) | 1.08(0.9-1.3) | 1.24(0.99-1.57) | 1.61(1.45-1.8) |

OR: Odds ratio. 95% CI: 95% confidence interval.
